# Association Between Phasic Vagal‐Mediated Heart Rate Variability and Momentary Exhaustion in Daily Life

**DOI:** 10.1002/smi.70074

**Published:** 2025-07-22

**Authors:** Magdalena Katharina Wekenborg, Christian Rominger, Andreas R. Schwerdtfeger

**Affiliations:** ^1^ Faculty of Medicine and University Hospital Carl Gustav Carus Else Kröner Fresenius Center for Digital Health TUD Dresden University of Technology Dresden Germany; ^2^ Department of Health Psychology Institute of Psychology University of Graz Graz Austria

**Keywords:** ambulatory assessment, exhaustion, heart rate variability, parasympathetic nervous system, stress, vagus

## Abstract

Stress‐related chronic exhaustion can be predicted longitudinally by reduced basic vagal tone (i.e., vagally‐mediated heart rate variability [vmHRV]). However, little is known about the relationship between phasic vmHRV and momentary exhaustion in daily life. To examine this relationship, this preregistered study used ecological momentary assessment (EMA) in a sample of *N* = 151 healthy participants (age = 22.17 years [SD = 4.98 years]; 14.57% male) for three consecutive weekdays. Exploratorily, we examined if individuals with higher chronic exhaustion would show different patterns of phasic vmHRV when perceiving acute stress. We analysed data on momentary (emotional, cognitive, physical) exhaustion, perceived acute stress, ambulatory ECG data and adjusted for relevant covariates (e.g., age, gender, and momentary movement acceleration) using multi‐level analyses. After adjusting for preregistered covariates, phasic vmHRV showed a positive association with momentary emotional and cognitive exhaustion, but not with momentary physical exhaustion. Our exploratory analyses revealed that individuals with higher levels of chronic exhaustion did not show the expected negative association between situationally perceived acute stress and phasic vmHRV, whereas those with lover levels did. These findings indicate that momentary exhaustion is associated with increased phasic vmHRV in daily life. Combined with our exploratory results that chronic exhaustion modulates vagal withdrawal under perceived acute stress, this study offers important directions for future research into the link between stress‐related exhaustion and autonomic changes.

**Study Registration:** The study and analysis plan were preregistered at OSF (DOI: 10.17605/OSF.IO/T2C4X).

## 1 Introduction

Chronic exhaustion, understood as a stress‐related phenomenon characterised by feelings of being depleted, is increasingly prevalent, as indicated by data from Sweden, the only country where exhaustion is recognised as a distinct diagnosis in the ICD‐10 (i.e., specification of F43.8 Reaction to severe stress, given the diagnostic code F43.8 A) (Socialstyrelsen 2024). In Sweden, the prevalence of exhaustion disorder has approached that of major depressive disorder (Höglund et al. 2020). This is particularly concerning given the consistent association between chronic exhaustion and cardiovascular diseases (CVD) risk (for review, see John et al. 2024), the primary cause of mortality worldwide (Roth et al. 2020).

As we (Wekenborg et al. 2023) and others (von Känel et al. 2023) have pointed out, sustained dysregulations of the autonomic nervous system (ANS) may be at least one explanation for the increased risk of CVD associated with chronic exhaustion, potentially mediated through corresponding metabolic and inflammatory changes (Wirtz and von Känel 2017). A special role seems to be played by dysregulations in the parasympathetic branch of the ANS as indicated by modulations in high‐frequency fluctuations in the time interval between heart beats (i.e., vagally‐mediated heart rate variability [vmHRV]) (Laborde et al. 2017; Porges 2007; Thayer and Lane 2000), an established risk factor for CVD (Thayer et al. 2010).

In a series of studies, we were among the first to show that higher chronic exhaustion was both cross‐sectionally (Kanthak et al. 2017) and longitudinally (Wekenborg, Hill, et al. 2019) associated with reduced baseline (i.e., tonic) vmHRV, operationalised as a seated 6 minutes resting condition with spontaneous breathing taken at one‐time point, a widely used short‐term measure of trait like cardiac vagal tone (Berntson et al. 1997; Bertsch et al. 2012; Task Force of the European Society of Cardiology and the North American Society of Pacing and Electrophysiology, 1996; Thayer and Lane 2009). These findings are in line with previous research on negative associations between chronic exhaustion (not necessarily within the framework of burnout) and tonic vmHRV, both as a short‐term measure (Lennartsson et al. 2016; Watanabe et al. 2002) and over longer periods (e.g., ≥ 24h) (Collins and Karasek 2010; Traunmüller et al. 2019; Zhang et al. 2018).

More importantly, our recent findings regarding the directionality of this association using cross‐lagged analyses, indicate that reduced vmHRV prospectively predicts higher chronic exhaustion, based on data collected within a population‐based sample over a period of 4 years, but not vice versa (Wekenborg et al. 2022). In sum, previous research indicates a potential role of modulations in tonic vmHRV as a biomarker for chronic exhaustion, opening a time window for prevention programs. In addition, these findings add to the understanding of the psychopathology of chronic exhaustion. Following the model of neurovisceral integration (NVI), these reductions in tonic vmHRV indicate disrupted communication between the frontal brain and body (Thayer and Lane 2009), impairing an individual's adequate and flexible response to environmental demands, potentially leading to even more severe pathologies (e.g., CVD) (Thayer et al. 2010).

Surprisingly, little research has so far investigated the relationship between state (i.e., phasic) vmHRV and momentary exhaustion in daily life at the within‐person level. In this study, we define momentary exhaustion as a transient state of fatigue lasting only a few hours or days, predictably occurring as a consequence of a preceding situational factor (Kluger et al. 2013). In contrast, chronic exhaustion, as understood here, manifests even at rest, without an immediately preceding stressor, and persists over an extended period (Schaufeli et al. 1996). More specifically, we conceptualise momentary exhaustion as a multidimensional construct comprising physical (bodily weakness or fatigue), emotional (emotional depletion and overwhelm), and cognitive (reduced concentration and mental fatigue) dimensions (Kluger et al. 2013; Schaufeli et al. 1996).

However, this seems particularly relevant since the effects observed at the between‐subject level cannot simply be applied to within‐person level relationships (Hamaker 2012). Especially with respect to vmHRV, previous research indicates that relationships between phasic vmHRV and psychological constructs are not as straightforward as those with tonic vmHRV (Laborde et al. 2017). Specifically, while higher values of tonic vmHRV are generally beneficial for individuals, the adaptiveness of phasic vmHRV values depends greatly on the specific characteristics of the situation (Mosley et al. 2017; Park et al. 2014). For example, during periods of exposure to a stressor, reduced cardiac vagal activity (indicated by lower vmHRV) is adaptive, as it facilitates the allocation of energy for coping with the stressor. In this context, our own previous research indicates that in individuals with high chronic exhaustion, this adaptive vmHRV reduction during confrontation with a standardised laboratory psychosocial stressor (i.e., Trier Social Stress Test [TSST]) (Kirschbaum et al. 1993) is absent (Wekenborg, von Dawans, et al. 2019). Further support for the idea that high vagal activity during perceived acute stress in individuals with high chronic exhaustion might hinder energy provision to cope with a stressor comes from a study by von Känel et al. (2023), which revealed associations between chronic exhaustion and reduced SNS reactivity (i.e., reduced epinephrine stress reactivity) during the TSST.

Understanding within‐person relationships between vmHRV and exhaustion appears to be of central importance, not only for a better fundamental understanding of underlying psychophysiological dynamics, but also for the development and evaluation of prevention and intervention programs. To date, to the best of our knowledge, only three studies simultaneously assessed phasic vmHRV and momentary exhaustion using ecological momentary assessment (EMA) methodology, all of which were published by the same research group (Schmid and Thomas 2020, 2021; Schmid et al. 2024).

Schmid and Thomas (2020) assessed 48h ECG‐data in a sample of school teachers during two typical school days. Momentary exhaustion was measured with six random prompts during each workday. Correlational analyses revealed no significant associations between phasic vmHRV (i.e., five‐minute vmHRV prior to each EMA questionnaire) and momentary exhaustion. Subsequently conducted multi‐level analyses for predicting momentary exhaustion did not include phasic vmHRV measures but only (i.e., tonic) vmHRV averaged over 48 h, which was significantly negatively predictive of momentary exhaustion (at level 2).

In a follow‐up study with health care professionals, the same authors (Schmid and Thomas 2021) using the same study design found no significant correlations between phasic vmHRV and momentary exhaustion. However, in this study, they used phasic vmHRV measures to predict momentary exhaustion and found that reduced phasic vmHRV was marginally significant in predicting higher momentary exhaustion during working shifts. Schmid et al. (2024) also simultaneously assessed phasic vmHRV and momentary emotional exhaustion. However, since their study focused on vmHRV stress reactivity, operationalised as within‐person differences in vmHRV in response to a stressor (i.e., time pressure), they did not report direct within‐person associations between phasic vmHRV and momentary emotional exhaustion. Instead, they report a significant positive relationship between vmHRV stress reactivity and momentary emotional exhaustion, meaning that a stronger decrease in phasic vmHRV in response to a stressor was associated with higher momentary emotional exhaustion. In this context, the study by Filosa et al. (2024) also appears relevant, as it employs a different methodology but still finds that momentary exhaustion is associated with changes in vmHRV. Specifically, they report a negative association between HRV measured during the workday and momentary exhaustion at the end of the day, suggesting that changes in HRV, even if not specifically phasic vmHRV, may be related to levels of momentary exhaustion. In sum, the few existing findings suggest that, consistent with previous research on tonic vmHRV and chronic exhaustion, phasic vmHRV may predict momentary exhaustion. A more comprehensive assessment of this relationship is needed. This includes not only a longer assessment period with more prompts but also the consideration of momentary exhaustion symptoms as a multidimensional construct (Billones et al. 2021). The EMA studies reported here focused on the burnout‐related exhaustion framework by Maslach et al. (2001), using items that do not differentiate among the various dimensions of exhaustion (i.e., emotional, cognitive, physical) (Schaufeli et al. 1996). As these dimensions may have different associations with phasic vmHRV, they should be assessed separately.

Therefore, the aim of this preregistered study was to simultaneously assess ambulatory ECG data and momentary exhaustion during three consecutive weekdays to test the following preregistered hypothesis:

H 1 (non‐directional) Individual variations in exhaustion symptoms are mirrored in variations in vmHRV in everyday life.

Regarding the direction of this relationship, the majority of studies report negative associations between tonic HRV and chronic exhaustion. As mentioned above, one cannot automatically infer relationships at the within‐subject level from relationships at the between‐subject level. Nevertheless, given the preliminary evidence suggesting a negative relationship between phasic vmHRV and momentary exhaustion (Schmid and Thomas 2021), we preregistered the following directional hypothesis while acknowledging the inherent uncertainty:

H 1.1 (directional) A decrease in vmHRV is accompanied by an approximately simultaneous increase in exhaustion symptoms.

To account for the repeatedly demonstrated influence of socio‐demographic and health‐related factors on associations between vmHRV and psychological constructs, we additionally preregistered the following hypothesis:

H 1.2 This association persists after adjustment for relevant covariates (e.g., age, sex, BMI, consumption patterns with respect to alcohol, caffeine, and nicotine, depressive symptoms, physical activity level).

As our previous research identified a lack of adaptive phasic vmHRV reduction during perceived acute stress in individuals with high chronic exhaustion in a laboratory setting, we aimed to explore whether this pattern also occurs in everyday life. Vagal withdrawal has been described as a prerequisite for effectively mobilising energy to cope with acute stressors (Thayer and Lane 2009). Together with findings by von Känel et al. (2023), which link chronic exhaustion to blunted SNS reactivity during perceived acute stress, this lack of vagal withdrawal may indicate impaired stress adaptation. To determine whether this laboratory‐based moderation effect of chronic exhaustion on phasic vmHRV during perceived acute stress extends to real‐life settings, we conducted an exploratory analysis in addition to our preregistered hypotheses.

## 2 Methods

### 2.1 Participants

This study was preregistered at the Open Science Framework (OSF) and included a sample of *N* = 151 university students (22 men). Participants were recruited at University of Graz, Austria (*n* = 62) and the TU Dresden, Germany (*n* = 89) via flyers distributed on campus and on social media platforms. The ethics committees of the University of Graz and the TU Dresden approved the study. We expected small within‐person effects as well as small‐to‐medium cross‐level interaction effects. The sample size ensured sufficient power (≥ 0.80) to detect these effect sizes at a significance level of 0.05 (Arend and Schäfer 2019). Inclusion criteria were an age of over 18 years, no diagnosed mental disorders within the past 6 months, and no cardiovascular diseases or medication affecting the cardiovascular system. The sample had a mean age of 22.17 years and a mean BMI of 21.59 kg/m2 (see Table 1). All participants gave written informed consent prior to the 3‐day assessment. Course credits were given as a compensation for their participation. Relevant data and customised syntax are available at OSF.

**TABLE 1:** Descriptives.

	Total sample	Graz	Dresden	t(p)/Chi^2(p)
N	151	89	62	
Gender	22 men	10 men	12 men	1.34 (0.247)
Age	22.17 (4.98)	21.60 (3.69)	23.00 (6.32)	1.78 (0.076)
BMI	21.59 (2.77)	21.30 (2.62)	22.00 (2.95)	1.54 (0.126)

Abbreviation: *BMI = Body Mass Index.*

### 2.2 Procedure

The present study included a brief laboratory session within the facilities at the University of Graz or the TU Dresden, followed by a 3‐day ambulatory assessment starting the day after laboratory session. On arrival, participants read and signed informed consent. Participants were then provided with an EcgMove4 sensor (movisens GmbH, Karlsruhe, Germany), which they wore using a costum‐build combination of chest straps and adhesive electrodes on their chest (by movisens GmbH; see e.g., A. R. Schwerdtfeger and Rominger (2024); A. R. Schwerdtfeger and Rominger 2024 for previous applications). They were then led to a separate room, where a five‐minute heart rate recording was taken while seated in a resting position, breathing spontaneously and looking at neutral pictures, which— as pre‐registered—is not considered in the analyses of the present study but reported to provide a complete overview of the study procedure. Afterwards, they answered a baseline questionnaire via the online survey application LimeSurvey (LimeSurvey GmbH, Hamburg, Germany; http://www.limesurvey.org). They received instructions for the study procedures during the subsequent ambulatory assessment, both orally and in writing, their appointment for returning the ECG devices, and were thanked before being dismissed. Self‐reports during the EMA procedure were collected via the Android‐exclusive movisensXS application (movisens GmbH, Karlsruhe, Germany). Participants without an Android smartphone were provided with a study device.

The ambulatory assessment lasted for 3 days following the lab session, which was conducted on weekdays, as previous research indicates differences in activity levels between weekdays and weekend (Zhong et al. 2008). Participants wore the ECG devices from 08:00–21:00 and received 10 random prompts a day with at least 30 min in between. Participants were instructed to complete a short self‐initiated questionnaire each evening during the assessment period. They rated how typical their day was compared to their usual days (‘Was today a typical weekday for you?’) and their level of physical activity (‘Have you been physically active today?’), both on a scale from 1 (‘not at all’) to 5 (‘very much’). Additionally, an open‐ended question allowed them to note anything they considered relevant.

### 2.3 Baseline Questionnaire

The baseline questionnaire included self‐report measures of sociodemographic and health relevant factors; only those relevant to the present study are described here. Age, gender, weight, and height (used to calculate the body mass index [BMI]) were assessed using self‐generated items. Chronic exhaustion was assessed using a German translation of the exhaustion subscale of the *Maslach Burnout Inventory ‐ General Survey* (MBI‐GS) (Schaufeli et al. 1996), as it is one of the most widely used measures in the field (Edú‐Valsania et al. 2022) and ensures comparability with our previous studies, in which we also applied the MBI‐GS (Kanthak et al. 2017; Wekenborg et al. 2022; Wekenborg, Hill, et al., 2019; Wekenborg, von Dawans, et al. 2019). The subscale consists of five items, for example, 'I feel emotionally drained from my work', each rated on a seven‐point Likert scale (0 = never; 6 = daily). To enhance specificity for university students, we added the following instruction: ‘Please refer to the activity that currently constitutes your main occupation (e.g., your studies, your job)’. The exhaustion subscale showed good reliability indicated by a Mc Donald's *ω* of 0.93. The mean chronic exhaustion score was *M* = 2.50 (SD = 1.33).

Depressive symptoms were due to the ongoing debate regarding the distinguishability between depressive symptoms and exhaustion. They were measured using the German version (PHQ‐D, Löwe et al. 2002) of the Patient Health Questionnaire (PHQ‐9, Kroenke et al. 2001), which comprises the nine diagnostic criteria for depressive disorder of the Diagnostic and Statistical Manual of Mental Disorders (American Psychiatric Association 2000), which are scored on a four‐point ranking scale (0 = not at all, 3 = nearly every day) and added up to a sum score (*M* = 15.40, SD = 3.66), which showed good Mc Donald's *ω* of 0.85.

### 2.4 Ambulatory Sensor

The EcgMove4 (movisens GmbH, Karlsruhe, Germany) continuously recorded the ECG via a modified Einthoven II‐lead (1024 Hz sampling rate) and acceleration information with the built‐in three‐dimensional accelero‐sensor (64 Hz sampling rate), as well as a barometric sensor (8 Hz sampling rate). The small device (62.3 mm × 38.6 × 11.5 mm) has a weight of 26 g and was worn at the chest.

### 2.5 Derived Ambulatory vmHRV Metrics

We calculated phasic vmHRV indices by means of the DataAnalyzer software (movisens GmbH, Karlsruhe, Germany). Within our analyses we concentrated on the most commonly used measure of vmHRV, specifically, the time‐domain measure root mean square of successive differences between successive R‐R intervals (RMSSD). RMSSD is an approved short‐term measure of vmHRV that reflects vagal cardiac influence. We used RMSSD because it has been recommended for ambulatory designs due to its cost‐effectiveness (Goedhart et al. 2007; Shaffer and Ginsberg 2017) and the relative robustness with respect to breathing patterns (e.g., Shaffer and Ginsberg 2017; Task Force of the European Society of Cardiology and the North American Society of Pacing and Electrophysiology, 1996; Hill et al. 2009). Furthermore, the majority of previous EMA studies on HRV have used RMSSD (Schmid and Thomas 2020, 2021; A. Schwerdtfeger and Friedrich‐Mai 2009; A. R. Schwerdtfeger and Rominger 2024) making it a suitable choice for embedding and interpreting our study in the context of other ambulatory research.

We focused on RMSSD scores 5 minutes preceding each prompt. Across all prompts, the mean RMSSD was 41.49 ms (SD = 26.08 ms), ranging from 2.24 to 272.17 ms. The mean RMSSD per person ranged between 14.95 and 121.93 ms (*M* = 41.74 ms, SD = 17.86 ms). We ln‐transformed the RMSSD score due to the skewed distribution (lnRMSSD). The ICC of the RMSSD was 0.39, indicating that 39% of the total variance is attributable to between‐person differences, leaving up to 61% as within‐person (and error) variance.

### 2.6 Movement Acceleration and Body Position

The sensor provides information about the movement acceleration (g) and body position in consecutive one‐minute segments. We used this information to calculate if the participant was in an upright or lying position. We used the mean of 5 minutes prior to each prompt for movement acceleration.

### 2.7 EMA Questions

In the absence of validated items to assess momentary emotional, cognitive, and physical exhaustion, we measured these key outcome variables at each prompt using the following self‐generated items: (1) ‘How emotionally exhausted did you feel in the last 5 min?’ (2) ‘How physically exhausted did you feel in the last 5 min?’ (3) ‘How cognitively exhausted did you feel in the last 5 min?’ Participants rated these variables using a five‐point Likert scale (1 = not at all; 5 = completely). The mean momentary emotionally exhaustion rating per prompt was 1.94 (SD = 0.99) with an *ICC* of 0.39, which indicates the proportion of variance attributed to stable between‐person (i.e., trait‐like) differences. For momentary physical exhaustion, the mean score was 2.16 (SD = 1.03, *ICC* = 0.37) and for momentary cognitive exhaustion the mean was 2.26 (SD = 1.03, *ICC* = 0.38). This indicates that up to 63% of the total variance might be due to within‐person (and error) variation. Additionally, participants were asked about their current location (at home [1779] vs. at work [890] vs. on transit [587]), nicotine (yes/no; 52 yes) and caffeine consumption (yes/no; 256 yes), and whether they felt nervous or stressed, all with respect to the last 5 minutes before the prompt. The ‘nervous or stressed’ item was rated using the same five‐point Likert scale as the main outcome variables (1 = not at all; 5 = completely; *M* = 2.03, SD = 0.98, *ICC* = 0.35). With respect to the 20 min preceding the prompt, participants were asked if they had consumed alcohol (yes/no; 56 yes), or engaged in physical activity (yes/no; 191 yes).

### 2.8 Deviations From the Pre‐Registration

First, deviating from the preregistration, we collected data not only in Graz but also in Dresden. Secondly, we included more participants (*N* = 151), than preregistered (*N* = 110), which provided us with greater statistical power to test our hypotheses. Third, we did not test our preregistered H2 regarding the use of specific patterns of non‐metabolic (additional) reductions in vmHRV (AddHRVr) to predict increases in situational exhaustion, to avoid exceeding the scope of this paper.

### 2.9 Available Data

We collected 559 self‐initialised prompts with a mean representativeness rating of *M* = 3.61 (SD = 1.02) and participants rated their sports activity as rather low (*M* = 2.28, SD = 1.24). Participants answered a total of 3715 random prompts over the three days. For each participant, the number of prompts ranged between three and 36 (*M* = 24.60, SD = 5.49). In total 114 alarms were dismissed, 947 alarms were ignored, and 82 alarms were incomplete. 3256 prompts of the 3715 answered prompts (87.64% of) were associated with valid vmHRV indices. No information was imputed, and we deleted prompts listwise if any information was missing.

### 2.10 Statistical Analyses

To predict momentary exhaustion (emotional, cognitive, and physical) via phasic vmHRV (i.e., lnRMSSD), we first calculated three separate multi‐level models where participants served as random factor and the person mean centred vmHRV (i.e., ln RMSSD) as fixed effects. We additionally controlled for age, gender, BMI, alcohol, caffeine, and nicotine consumption, depressive symptoms, momentary movement acceleration (g). We preregistered these control variables as they are well known to influence phasic vmHRV in EMA settings (see e.g., A. Schwerdtfeger and Friedrich‐Mai 2009; A. R. Schwerdtfeger and Rominger 2024). Preliminary own findings indicate a potential moderation effect of chronic exhaustion on vmHRV stress‐reactivity within a laboratory setting (Wekenborg, von Dawans, et al. 2019). In order to examine whether this moderation effect would also be visible within daily life, we conducted an exploratory (i.e., not pre‐registered) evaluation to determine whether individuals with higher chronic exhaustion would show a different pattern of phasic vmHRV when perceiving acute stress by calculating a model with participants as random factor and chronic exhaustion, person mean‐centred perceived acute stress, and their interaction as predictors. The phasic lnRMSSD served as criterion variable. For all MLMs, participants served as random factor and all other variables were treated as fixed effects.

For all analyses, we accounted for autoregression and used R (version 4.2.0; R Core Team 2023) with the package nlme (version 3.1–162, Pinheiro et al. 2023). To assess moderation effects, we calculated simple slopes using the reghelper package (version 1.1.1, Hughes and Beiner 2022). The assumptions underlying multilevel models such as linearity, normality of residuals, homoscedasticity, and multicollinearity among the predictors were evaluated.

## 3 Results

### 3.1 Results for Preregistered Hypotheses

The results regarding the testing of our three preregistered hypotheses are presented in Table 1. In line with H1, phasic lnRMSSD predicted momentary emotional, cognitive, and physical exhaustion. In line with H1.1 regarding the directionality of this association, phasic lnRMSSD was negatively linked with momentary physical exhaustion (*b* = −0.07, *t* (3104) = −2.492, *p* = 0.013). However, contrary to our expectations, it was positively associated with momentary emotional (*b* = 0.06, *t* (3104) = 2.122, *p* = 0.034) and momentary cognitive exhaustion (*b* = 0.10, *t* (3104) = 3.390, *p* < 0.001). Regarding H1.2, the positive link between phasic lnRMSSD and momentary emotional (*b* = 0.07, *t* (3100) = 2.219, *p* = 0.027) and momentary cognitive exhaustion (*b* = 0.08, *t* (3100) = 2.429, *p* = 0.015) remained after adjusting for the preregistered covariates. However, the negative link between phasic lnRMSSD and momentary physical exhaustion shrunk (*b* = −0.04, *t* (3100) = −1.061, *p* = 0.289). This reduction was due to the observation that movement acceleration (g) before the prompt was positively associated with momentary physical exhaustion (*b* = 0.62, *t* (3104) = 2.882, *p* = 0.004) as well as negatively with phasic lnRMSSD (*b* = −3.89, *t* (3104) = −34.96, *p* < 0.001). Thus, after adjusting for movement acceleration (g), the effect of phasic lnRMSSD on momentary physical exhaustion disappeared [Table 2]. Adding additional control variables such as momentary safety, location (at home vs. at work vs. on transit), body position (upright vs. sitting vs. lying), self‐reported speaking (0 = no, 1 = yes), and social environment (0 = no, 1 = yes) did not change any of the results (Supporting Information S1: Table S1).

**TABLE 2:** Three multi‐level models predicting the three dimensions of momentary exhaustion.

	Emotion model			Cognitive model			Physical model		
Predictors	*b*	Conf. Int (95%)	*p*‐value	*b*	Conf. Int (95%)	*p*‐value	*b*	Conf. Int (95%)	*p*‐value
Intercept	0.18	−1.25–1.62	0.801	0.87	−0.68–2.41	0.270	1.50	−0.05–3.05	0.059
Phasic vmHRV (lnRMSSD)	0.07	0.01–0.14	**0.027**	0.08	0.02–0.15	**0.015**	−0.04	−0.10–0.03	0.289
Tonic vmHRV (lnRMSSD)	−0.09	−0.32–0.14	0.434	−0.02	−0.26–0.22	0.873	−0.13	−0.37–0.11	0.295
Physical activity	0.16	−0.30–0.63	0.487	−0.23	−0.72–0.26	0.356	0.42	−0.07–0.92	0.092
Age	0.01	−0.01–0.02	0.582	−0.00	−0.02–0.02	0.666	0.00	−0.02–0.02	0.917
Gender (0 = women, 1 = men)	0.13	−0.13–0.39	0.325	−0.04	−0.31–0.24	0.792	−0.04	−0.31–0.24	0.801
BMI	0.01	−0.02–0.04	0.482	0.01	−0.02–0.05	0.543	−0.00	−0.04–0.03	0.914
Alcohol (0 = no, 1 = yes)	−0.33	−0.54–−0.12	**0.002**	−0.17	−0.39–0.05	0.134	−0.00	−0.23–0.22	0.982
Caffeine (0 = no, 1 = yes)	−0.11	−0.21–−0.00	**0.046**	−0.14	−0.25–−0.03	**0.015**	−0.18	−0.29–−0.07	**0.002**
Nicotine (0 = no, 1 = yes)	0.22	−0.02–0.46	0.069	0.02	−0.24–0.27	0.906	0.24	−0.02–0.49	0.071
Depressive symptoms	0.10	0.07–0.12	**<** **0.001**	0.09	0.07–0.12	**<** **0.001**	0.08	0.05–0.11	**<** **0.001**
Random effects									
σ^2^	0.59			0.66			0.67		
*τ*_00_	0.27 _Participant_			0.31 _Participant_			0.31 _Participant_		
ICC	0.31			0.32			0.32		
N	151 _Participant_			151 _Participant_			151 _Participant_		
Observations	3256			3256			3256		
Marginal *R* ^2^/Conditional *R* ^2^	0.137/0.405			0.114/0.396			0.090/0.379		

*Note:* Bold values denotes the *p*‐values of the corresponding b coefficients.

Abbreviations: BMI = Body Mass Index; lnRMSSD = root mean square of successive differences between successive R‐R intervals, logarithmized; vmHRV = vagally‐mediated heart rate variability.

### 3.2 Results of Exploratory Analyses

**FIGURE 1:**
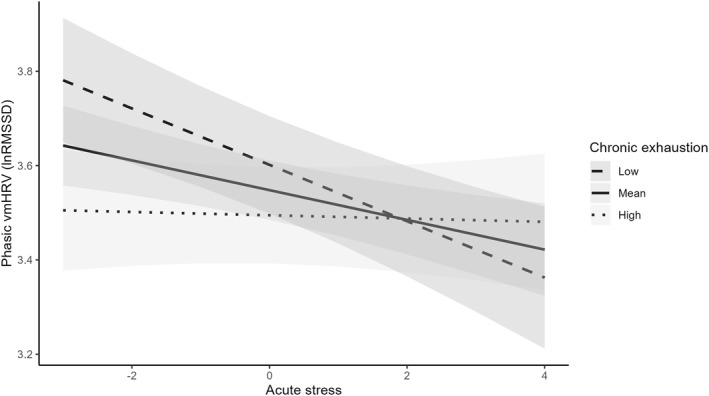
Simple slopes of acute stress predicting phasic vagally‐mediated heart rate variability (vmHRV), operationalised as root mean square of successive differences between successive R‐R intervals, logarithmized (lnRMSSD), for low and high levels of chronic exhaustion. High levels represent *M* + 1 SD, low levels represent *M* −1 SD.

The analyses investigating the effects of perceived acute stress and chronic exhaustion on phasic lnRMSSD, and adjusting for gender, age, alcohol, nicotine, and caffeine consumption, as well as BMI, movement (g), and depressive symptoms, indicated a significant interaction between chronic exhaustion and perceived acute stress (*b* = 0.02, *t* (3099) = 2.96, *p* = 0.003). Following the simple slope analyses, we found that only participants with low levels of chronic exhaustion (−1SD) showed the expected negative association between perceived acute stress and phasic lnRMSSD (*b* = −0.06, *t* (3099) = −4.28, *p* < 0.001), whereas individuals with higher levels of chronic exhaustion (+ 1SD) showed no significant link between perceived acute stress and phasic lnRMSSD (*b* = −0.00, *t* (3099) = −0.26, *p* = 0.794; see Figure 1). Again, adding additional covariates did not alter the results (Supporting Information S1: Table S2).

## 4 Discussion

This preregistered study investigated the relationship between phasic vmHRV and momentary exhaustion in everyday life. As hypothesised (H1), we found a significant association between these variables. Specifically, while phasic RMSSD was negatively linked to momentary physical exhaustion, as expected (H1.1), it showed an unexpected positive association with momentary emotional and cognitive exhaustion—an effect that persisted even after adjusting for the preregistered covariates (H1.2). This finding initially seems surprizing as, to the best of our knowledge, all previous studies that have reported significant relationships between tonic vmHRV and chronic exhaustion consistently found that, at the between‐person level, higher tonic vmHRV is associated with reduced chronic exhaustion (Kanthak et al. 2017; Lennartsson et al. 2016; Watanabe et al. 2002; Wekenborg, Hill, et al., 2019; Wekenborg et al., 2022; Zhang et al. 2018). Upon closer examination, however, our finding highlights that associations between variables can differ significantly at the between‐person and within‐person levels. This underscores the necessity of examining these relationships separately rather than generalising from one level to the other (Hamaker 2012).

Our finding of a positive association between phasic vmHRV and momentary (cognitive and emotional) exhaustion is also unexpected, considering that, currently, the only study that has reported significant effects in this relationship found negative associations (Schmid and Thomas 2021). However, it should be noted regarding the study by Schmid and Thomas (2021) that the negative association reported was not reliable (i.e., *p* = 0.07).

Additionally, unlike our study, their studies measured exhaustion solely as emotional exhaustion using a single item, without considering cognitive and physical aspects. Our divergent results for momentary physical, emotional, and cognitive exhaustion underscore the repeatedly emphasised importance of accounting for the multidimensionality of the exhaustion construct (Billones et al. 2021) and suggest that individuals can differentiate between these three dimensions, at least with regard to momentary exhaustion. Specifically, we observed negative relationships between phasic vmHRV and momentary physical exhaustion, while positive relationships were found with momentary emotional and momentary cognitive exhaustion. Given this pattern of results, the following considerations can be made: First, momentary physical exhaustion may simply be a consequence of physical activity, and when controlled for this variable, the relationship with phasic vmHRV disappears. Second, this finding suggests that, with regard to associations with phasic vmHRV, momentary emotional and cognitive exhaustion dimensions are more psychologically relevant and should receive more attention in future research.

Given the limited findings in this area, it remains unclear whether the positive relationship between phasic vmHRV and momentary exhaustion observed in a single study reflects a consistent pattern. Before drawing further conclusions, additional studies are needed to establish the robustness of this association, particularly with more socio‐demographically diverse study samples. Nevertheless, the question arises as to how this pattern can be explained and what its implications are.

Broadly speaking, one could argue that since exhaustion can be viewed as an indicator of low energetic arousal, observing high phasic vmHRV is a plausible physiological response, as it reflects elevated parasympathetic activity, which typically occurs during states of low arousal, such as relaxation (Renna et al. 2020). Unlike relaxation, which is typically accompanied by positive feelings (Benson et al. 1975), exhaustion is, by definition, an aversive condition (Billones et al. 2021; Maslach et al. 2001). Therefore, in this case, higher phasic vmHRV is associated with an aversive state, highlighting the importance of considering psychological states when measuring phasic vmHRV to ensure proper contextual understanding of the results (A. R. Schwerdtfeger and Rominger 2024).

Regarding the adaptiveness of the revealed pattern, as highlighted by Laborde et al. (2017), phasic levels of vmHRV are more complex to interpret than tonic vmHRV levels. Apart from a few exceptions (Peschel et al. 2016; Stein et al. 2005), literature consistently shows that higher tonic vmHRV, reflecting increased resting cardiac vagal activity, is beneficial in most situations, whereas low tonic vmHRV is associated with compromised health and disease (Thayer et al. 2012). In contrast, the adaptiveness of high levels of phasic vmHRV strongly depends on the specific situation and its demands. Thus, increased phasic vmHRV appears more advantageous in the absence of immediate stressors, where recovery should be the focus, while reduced phasic vmHRV can be necessary when managing internal or external stressors, as it provides the organism with the required energy for the situation (Laborde et al. 2017).

Our results do not clarify whether the observed increased vmHRV is an adaptive response for energy restoration to overcome momentary exhaustion or a maladaptive mechanism reflecting a state of low motivation, which could hinder the individual's ability to mobilise energy for meeting demands. Supporting the notion of an adaptive function of this pattern, one could argue that since momentary exhaustion indicates a temporary overload of stress‐regulating systems after phases of increased external or internal demands in daily life, increased phasic vmHRV serves as an adaptive mechanism by facilitating the replenishment of depleted energy resources and thereby enhancing the individual's ability to cope with future stressor confrontations. Conversely, increased phasic vmHRV in the context of momentary exhaustion could reflect insufficient vagal withdrawal, which may impair the individual's ability to mobilise energy and cope with future stressors. To better understand this proposed maladaptive mechanism, it is important to keep in mind that a temporary reduction in vagal activity is essential for successful coping with stressors, as it enhances sympathetic activation necessary for energy mobilisation (Chrousos 2009), even though it should be noted that the interactions between PNS and SNS during perceived acute stress are complex (Weissman and Mendes 2021). Thus, an increased vmHRV might indicate hampered vagal withdrawal, potentially impeding effective energy mobilisation and signalling increased disengagement (or attenuated engagement) when feeling emotionally and cognitively exhausted.

Experimental studies manipulating phasic vmHRV during momentary exhaustion appear to be a suitable approach for assessing its adaptive function. If this pattern indeed represents an adaptive mechanism for energy restoration following phases of increased external or internal demands, an experimentally induced additional increase in vmHRV (e.g., through breathing exercises; e.g., A. R. Schwerdtfeger et al. 2020) should further accelerate the replenishment of energy resources, ultimately leading to improved coping ability when facing subsequent stressors. Conversely, if increased phasic vmHRV in the context of momentary exhaustion reflects a maladaptive mechanism, an experimentally induced further increase in vmHRV should result in impaired coping with future stressors.

Initial evidence for an exhaustion‐associated impeded effective energy mobilisation in the face of perceived acute stress exposure associated with insufficient vagal withdrawal comes from our own previous research on chronic exhaustion, even though these findings cannot be directly generalised to momentary exhaustion: We found that individuals with higher chronic exhaustion symptoms showed increased phasic vmHRV in response to a standardised laboratory psychosocial stressor (i.e., TSST) (Kirschbaum et al. 1993) compared to those with lower chronic exhaustion symptoms, who exhibited reduced phasic vmHRV (Wekenborg, von Dawans, et al. 2019). This idea is further supported by a recently published study of von Känel et al. (2023), which revealed that higher levels of chronic exhaustion were associated with reduced epinephrine increases pre‐compared to post‐TSST exposure, indicating a blunted sympathetic nervous system (SNS) response to perceived acute stress. These effects were specific to epinephrine and did not manifest in other SNS markers, such as heart rate and salivary alpha‐amylase, which explains why we did not observe these exhaustion‐associated effects in our previous study, as we used exactly these markers (Wekenborg, von Dawans, et al. 2019). However, no conclusions on the role of phasic vagal activity for this effect can be drawn as von Känel et al. (2023) did not report vmHRV data in this paper.

To further validate our idea of a potential role of chronic exhaustion‐associated augmentation in phasic vmHRV during perceived acute stress, we exploratively (i.e., not pre‐registered) examined if chronic exhaustion would moderate associations between perceived acute stress and vmHRV in everyday life. Our exploratory analyses indicated that only individuals with low chronic exhaustion showed the expected vagal withdrawal (i.e., reduced phasic vmHRV) during situations perceived as stressful, supporting our idea of a chronic exhaustion‐associated reduced flexibility in stress reactivity.

Given the non‐preregistered nature of these analyses, caution is warranted in interpreting these findings. However, our results align with blunted stress responses observed in other mental disorders that share significant symptomatic overlap with chronic exhaustion, particularly depressive symptoms (Carroll et al. 2007; Phillips 2011; Phillips et al. 2011). This supports the plausibility of our findings, yet they should still be interpreted with caution. Hypothesis‐driven further empirical validation using comprehensive assessments of psychophysiological responses to perceived acute stress is therefore essential, particularly in individuals with varying levels of chronic exhaustion. Understanding the psychophysiological mechanisms underlying the observed modulations in vagal withdrawal associated with chronic exhaustion during perceived acute stress, as well as their consequences for stress reactivity of the two main energy‐mobilising systems ‐the SNS and the Hypothalamic‐Pituitary‐Adrenal axis ‐ during perceived acute stress, appears highly relevant, as blunted stress responses have been associated with adverse behavioural and health outcomes (Carroll et al. 2017). If future empirical studies confirm the blunted physiological stress response associated with chronic exhaustion and provide a deeper understanding of the psychophysiological mechanisms, changes in stress reactivity could potentially be used as a diagnostic tool in clinical settings and may also help inform prevention and intervention strategies.

It is important to note that, in line with our hypothesis, all effects found in the present study remained significant even after adjusting for depressive symptoms. These findings challenge the idea that in general, effects found for chronic exhaustion symptoms operationalised within the burnout framework, merely reflect underlying depressive symptoms, as often suggested (Bianchi et al. 2015). Instead, our findings suggest, in line with our previous research (Kanthak et al. 2017; Wekenborg, Hill, et al., 2019; Wekenborg et al., 2022), independent modulations of vmHRV specific to exhaustion.

### 4.1 Limitations

The present research has several limitations. First, due to the absence of a universally accepted definition of momentary and chronic exhaustion, we used self‐generated items to assess momentary exhaustion and the MBI‐GS exhaustion subscale to measure chronic exhaustion. While self‐generated items and the MBI‐GS have methodological limitations (e.g., Bouman et al. 2002; Demerouti 2006), we believe that this represents the most viable methodological approach available at this time.

Second, our study exclusively involved healthy individuals, which restricted the variance of clinically relevant exhaustion symptoms, both momentary and chronic. Together with the fact that our sample was primarily composed of university students, who may differ from the general population in terms of age, life experiences, and stress profiles, the result may have limited generalisability.

Third, data were collected at two locations (i.e., Graz and Dresden), which was not part of our preregistered protocol. While statistical analyses indicated no significant differences between the samples on key sociodemographic variables, potential differences in other factors cannot be ruled out. Nonetheless, despite the potential limitations of including data from two different (but same language) countries, we believe that this approach enhances the external validity of our study and supports the robustness of our results.

Fourth, although we adjusted for key potential confounders, other factors (i.e., prior illnesses, habitual physical activity, medication use) may have also influenced the observed effects.

Fifth, the EMA is a correlative approach, so causal inferences cannot be made.

## 5 Conclusions

In concluding, this preregistered study is the first to demonstrate that (by movisens GmbH; see e.g., A. R. Schwerdtfeger and Rominger (2024); A. R. Schwerdtfeger and Rominger 2024 for previous applications). In line with our preregistered hypothesis H1.1, we observed a negative association between phasic vmHRV and acute physical exhaustion. However, this effect did not remain after adjusting for relevant covariates, including depressive symptoms. In contrast, positive associations with acute emotional and cognitive exhaustion remained robust, though their direction was not expected. These results highlight that acute exhaustion dimensions differ in their physiological correlates and that phasic vmHRV does not uniformly reflect lower acute exhaustion. Additionally, our exploratory analysis supports laboratory findings that chronic exhaustion is linked to modulation of cardiac vagal withdrawal under perceived acute stress in everyday life settings. Future research should focus on elucidating the mechanisms behind the increase in phasic vmHRV with momentary exhaustion and the chronic exhaustion‐related modulation of vagal withdrawal in response to perceived acute stress, as well as exploring potential connections between these processes.

## Ethics Statement

Ethics committees of the University of Graz (GZ. 39/80/63 ex 2022/22) and the TU Dresden (GZ. SR‐EK‐452102023).

## Consent

Informed consent was obtained from all individual participants included in the study.

## Conflicts of Interest

The authors declare no conflicts of interest.

## Supporting information

Supporting Information S1

## Data Availability

De‐identified data from this study are available in a public archive (https://osf.io/t2c4x/files/osfstorage). Materials availability. Materials used to conduct the study are not publicly available.
