# Supplementary material for: Association Between Phasic Vagal‐Mediated Heart Rate Variability and Momentary Exhaustion in Daily Life
Source: Stress Health. 2025 Jul 22;41(4):e70074. doi: 10.1002/smi.70074 (PMC12282498; doi:10.1002/smi.70074)
Supplement: Supplementary file 1 — Supporting Information S1 [file SMI-41-e70074-s001.docx]

Table S1. Three multi-level prediction models predicting the three facets of acute exhaustion with additional control variables

|  | **Emotion model** | | | **Cognitive model** | | | **Physical model** | | |
| --- | --- | --- | --- | --- | --- | --- | --- | --- | --- |
| *Predictors* | *b* | *Conf. Int (95%)* | *p-value* | *b* | *Conf. Int (95%)* | *p-value* | *b* | *Conf. Int (95%)* | *p-value* |
| Intercept | 2.74 | 1.27 – 4.21 | **<0.001** | 2.89 | 1.20 – 4.57 | **0.001** | 3.28 | 1.55 – 5.00 | **<0.001** |
| Phasic vmHRV (lnRMSSD) | 0.09 | 0.02 – 0.15 | **0.008** | 0.09 | 0.02 – 0.16 | **0.009** | -0.03 | -0.10 – 0.04 | 0.447 |
| Tonic vmHRV (lnRMSSD) | -0.03 | -0.23 – 0.17 | 0.774 | 0.02 | -0.21 – 0.25 | 0.887 | -0.10 | -0.34 – 0.13 | 0.393 |
| Physical activity | 0.21 | -0.31 – 0.73 | 0.434 | 0.18 | -0.37 – 0.73 | 0.525 | 0.18 | -0.39 – 0.75 | 0.541 |
| Age | 0.00 | -0.01 – 0.02 | 0.563 | -0.00 | -0.02 – 0.01 | 0.670 | 0.00 | -0.02 – 0.02 | 0.858 |
| Gender (0 = women, 1 = men) | 0.19 | -0.04 – 0.41 | 0.103 | 0.02 | -0.24 – 0.28 | 0.877 | 0.02 | -0.25 – 0.28 | 0.897 |
| BMI | 0.01 | -0.02 – 0.04 | 0.382 | 0.01 | -0.02 – 0.05 | 0.461 | -0.00 | -0.03 – 0.03 | 0.957 |
| Alcohol (0 = no, 1 = yes) | -0.31 | -0.51 – -0.10 | **0.003** | -0.10 | -0.32 – 0.12 | 0.376 | 0.03 | -0.20 – 0.25 | 0.799 |
| Caffeine (0 = no, 1 = yes) | -0.09 | -0.19 – 0.01 | 0.066 | -0.14 | -0.24 – -0.03 | **0.013** | -0.13 | -0.24 – -0.02 | **0.018** |
| Nicotine (0 = no, 1 = yes) | 0.23 | 0.00 – 0.46 | **0.048** | 0.03 | -0.21 – 0.28 | 0.809 | 0.26 | 0.01 – 0.52 | **0.040** |
| Depressive symptoms | 0.05 | 0.03 – 0.08 | **<0.001** | 0.05 | 0.03 – 0.08 | **<0.001** | 0.05 | 0.02 – 0.08 | **0.001** |
| Safety (Level 1) | -0.36 | -0.40 – -0.32 | **<0.001** | -0.33 | -0.37 – -0.28 | **<0.001** | -0.12 | -0.17 – -0.08 | **<0.001** |
| Safety (Level 2) | -0.51 | -0.66 – -0.35 | **<0.001** | -0.41 | -0.59 – -0.24 | **<0.001** | -0.31 | -0.49 – -0.13 | **0.001** |
| Location (at home vs. at work) | -0.03 | -0.11 – 0.05 | 0.441 | 0.06 | -0.03 – 0.15 | 0.164 | -0.02 | -0.11 – 0.07 | 0.718 |
| Location (at home vs. on transit) | 0.03 | -0.06 – 0.13 | 0.456 | -0.03 | -0.13 – 0.07 | 0.516 | 0.10 | -0.00 – 0.20 | 0.056 |
| Body Position (upright vs. sitting) | -0.01 | -0.10 – 0.09 | 0.902 | 0.09 | -0.01 – 0.19 | 0.077 | -0.26 | -0.36 – -0.15 | **<0.001** |
| Body Position (upright vs. lying) | -0.09 | -0.21 – 0.03 | 0.128 | -0.03 | -0.16 – 0.09 | 0.585 | -0.20 | -0.32 – -0.07 | **0.003** |
| Speak (0 =no, 1 = yes) | 0.01 | -0.05 – 0.08 | 0.701 | -0.08 | -0.15 – -0.01 | **0.025** | -0.16 | -0.23 – -0.09 | **<0.001** |
| Social environment (0 =no, 1 = yes) | -0.04 | -0.12 – 0.04 | 0.307 | 0.01 | -0.07 – 0.10 | 0.739 | 0.06 | -0.03 – 0.15 | 0.203 |
| **Random Effects** | | | | | | | | | |
| σ^2^ | 0.54 | | | 0.61 | | | 0.65 | | |
| τ_00_ | 0.20 _Participant_ | | | 0.27 _Participant_ | | | 0.28 _Participant_ | | |
| ICC | 0.27 | | | 0.31 | | | 0.30 | | |
| N | 151 _Participant_ | | | 151 _Participant_ | | | 151 _Participant_ | | |
| Observations | 3256 | | | 3256 | | | 3256 | | |
| Marginal R^2^ / Conditional R^2^ | 0.254 / 0.456 | | | 0.196 / 0.441 | | | 0.132 / 0.394 | | |

Note. *BMI = Body Mass Index; vmHRV = vagally-mediated heart rate variability; lnRMSSD =* root mean square of successive differences between successive R-R intervals, logarithmized

Table S2. Multi-level model predicting lnRMSSD via phasic stress and chronic exhaustion and their interaction as well as additional control variables

| *Predictors* | *b* | *Conf. Int (95%)* | *p-value* |
| --- | --- | --- | --- |
| Intercept | 4.08 | 3.06 – 5.10 | **<0.001** |
| Phasic stress | -0.06 | -0.10 – -0.02 | **0.003** |
| Chronic exhaustion | -0.03 | -0.09 – 0.03 | 0.329 |
| Phasic physical activity | -2.25 | -2.53 – -1.98 | **<0.001** |
| Age | -0.01 | -0.03 – 0.00 | 0.073 |
| Gender (0 = women, 1 = men) | -0.08 | -0.27 – 0.10 | 0.374 |
| BMI | -0.01 | -0.03 – 0.01 | 0.476 |
| Alcohol (0 = no, 1 = yes) | -0.05 | -0.16 – 0.06 | 0.385 |
| Caffeine (0 = no, 1 = yes) | 0.01 | -0.04 – 0.07 | 0.657 |
| Nicotine (0 = no, 1 = yes) | -0.04 | -0.16 – 0.09 | 0.587 |
| Depressive symptoms | 0.01 | -0.01 – 0.03 | 0.405 |
| Phasic safety (Level 1) | 0.02 | -0.01 – 0.04 | 0.200 |
| Tonic safety (Level 2) | 0.06 | -0.07 – 0.19 | 0.340 |
| Location (at home vs. at work) | -0.02 | -0.07 – 0.02 | 0.287 |
| Location (at home vs. on transit) | -0.15 | -0.21 – -0.10 | **<0.001** |
| Body Position (upright vs. sitting) | -0.20 | -0.25 – -0.15 | **<0.001** |
| Body Position (upright vs. lying) | -0.50 | -0.56 – -0.43 | **<0.001** |
| Speak (0 = no, 1 = yes) | 0.04 | 0.01 – 0.08 | **0.017** |
| Social environment (0 = no, 1 = yes) | 0.00 | -0.04 – 0.05 | 0.892 |
| Phasic stress x chronic exhaustion | 0.02 | 0.00 – 0.03 | **0.012** |
| **Random Effects** | | | |
| σ^2^ | 0.16 | | |
| τ_00_ _Participant_ | 0.14 | | |
| ICC | 0.47 | | |
| N _Participant_ | 151 | | |
| Observations | 3256 | | |
| Marginal R^2^ / Conditional R^2^ | 0.244 / 0.600 | | |
